# A description of the volume and intensity of sporadic physical activity among adults

**DOI:** 10.1186/2052-1847-7-2

**Published:** 2015-01-10

**Authors:** Jordan Robson, Ian Janssen

**Affiliations:** School of Kinesiology and Health Studies, Queen’s University, Kingston, ON K7L 3N6 Canada; Department of Public Health Sciences, Queen’s University, Kingston, ON K7L 3N6 Canada

**Keywords:** Motor activity, Health surveys, Adult

## Abstract

**Background:**

Emerging evidence indicates that accumulating physical activity in periods of less than 10 minutes, termed sporadic physical activity (SPA), has similar effects on health as a similar volume of bouted physical activity (BPA). The purpose of this study was to describe the volume and intensity of SPA in adults.

**Methods:**

Participants consisted of a representative sample of 6040 adults aged 20 years and older from the 2003–2006 U.S. National Health and Examination Nutrition Survey. Physical activity was measured over 7 days using Actigraph AM-7164 accelerometers. Each minute of accelerometer data was initially categorized by intensity (sedentary, light, moderate-to-vigorous), and then non-sedentary time was categorized as following a BPA or SPA pattern (≥ or < 10 consecutive minutes).

**Results:**

American adults accumulated 103 minutes/day of SPA of an intensity, which represented 27% of their total (BPA + SPA) daily physical activity. Only 3 minutes/day of the SPA was of a moderate-to-vigorous intensity; however, participants accumulated 16 minutes/day of moderate-to-vigorous activity embedded within light intensity BPA. This embedded moderate-to-vigorous activity represented 85% of total daily moderate-to-vigorous activity.

**Conclusions:**

SPA accounted for about a quarter of total daily physical activity. While the amount of moderate-to-vigorous SPA was minimal, a significant amount of moderate-to-vigorous activity was accumulated within bouts of primarily light intensity activity.

## Background

Physical inactivity is common in the modern Western lifestyle [1], where it plays a role in the development of several chronic diseases [2]. Public health guidelines for physical activity are that adults accumulate at least 150 minutes per week of moderate-to-vigorous intensity physical activity (MVPA) in bouts of 10 minutes or more [3–5]. This guideline is met by less than 15% of North American adults [6, 7]. While sporadic physical activity (SPA), or physical activity occurring in periods of less than 10 consecutive minutes, is not recognized as providing health benefits within the current physical activity guidelines, a growing body of evidence suggests otherwise. Specifically, sporadic MVPA is associated with cardiometabolic risk factors independent of bouted MVPA [8–10], and the associations between SPA and cardiometabolic risk factors are as strong as they are for bouted physical activity (BPA) [10]. Notable barriers to physical activity participation, including a lack of time and self-efficacy [11–13], may be less relevant for SPA than BPA.

Very little is known about how much SPA people accumulate. Data from a representative sample of adolescents [14] and a small convenience sample of older adults [15] suggest that approximately 66% of MVPA occurs sporadically. However, the relative time spent engaging in moderate or vigorous intensity SPA is unknown. Additionally, no descriptive information exists about the amount of time spent engaging in light intensity SPA. There is a growing body of evidence indicating that light intensity physical activity provides health benefits independent of MVPA [16, 17]. Thus, it is important to consider all movement intensities in studies of physical activity and not just MVPA.

The purpose of this study is to describe SPA in adults. Specifically, this study describes how much daily SPA adults accumulate, the intensity at which it is accumulated, the contribution of SPA to total physical activity (ie, SPA + BPA), and whether these characteristics vary by sociodemographic factors. These results provide foundational knowledge that could be used to help design SPA interventions.

## Methods

### Study design and participants

Participants were from the 2003–2004 and 2005–2006 cycles of the U.S. National Health and Nutrition Examination Survey (NHANES), a nationally representative cross-sectional survey [18]. NHANES collects data through home interviews and physical examinations in mobile exam centers. All participants gave informed consent and NHANES was granted ethics approved by the National Center for Health Statistics.

The current study was limited to NHANES participants aged 20 years of age and older, non-pregnant women, and those who completed both the home interview and mobile exam center visit. This left an eligible sample of 9451. We excluded 3411 participants with missing or invalid physical activity accelerometer data (explained below), leaving a final sample of 6040.

Potential selection bias arising from the lack of valid physical activity accelerometer data in 36% of eligible participants was assessed by comparing several characteristics (sex, age, ethnicity, obesity) between the 6040 participants with valid data to the 3411 without. Differences were observed between the two samples for age, sex, and ethnicity but not for obesity. The age, sex, and racial differences were used to derive new sample weights to account for the selection bias.

### Sociodemographic variables

Sociodemographic variables included sex, age (20–39 years old, 40–59 years old or 60+ years old), race/ethnicity (non-Hispanic white, non-Hispanic black, Mexican American or other) and body mass index (BMI) status (underweight <18.5 kg/m^2^, normal weight 18.5-24.9 kg/m^2^, overweight 25–29.9 kg/m^2^, or obese ≥30 kg/m^2^[19]). BMI values were calculated from measured heights and weights.

### Physical activity

SPA and BPA were assessed using Actigraph AM-7164 uniaxial accelerometers (Actigraph, Ft. Pensacola, FL). These accelerometers recorded average movement intensity, measured by counts in 1-minute intervals or epochs. Participants were given accelerometers at their mobile examination center visit and asked to wear them on an elasticized belt on their right hip for the 7 days after the visit. Participants were instructed to only remove the accelerometer when sleeping or when the accelerometer would get wet (e.g., bathing or swimming). After the 7 day measurement period was completed, participants mailed the accelerometers back to the NHANES researchers. The accelerometers were then tested to ensure calibration, the data were downloaded, and implausible count values were removed from the accelerometer database.

Further data reduction of the accelerometer database was carried out by the authors based on existing protocols [10, 20–24]. Initially, we removed non-wear periods and calculated the wear time for each day. Non-wear periods were defined as periods with ≥ 90 minutes of zero counts, with an allowance for 2 minutes of counts between 0 and 100 [22]. Next, each day was coded as valid or invalid and invalid days were removed from the dataset. Days were considered valid if the participant had ≥ 10 hours of wear time [20, 21, 24]. We then removed participants with 3 or fewer valid days [20, 21, 23, 24].

**Figure 1:**
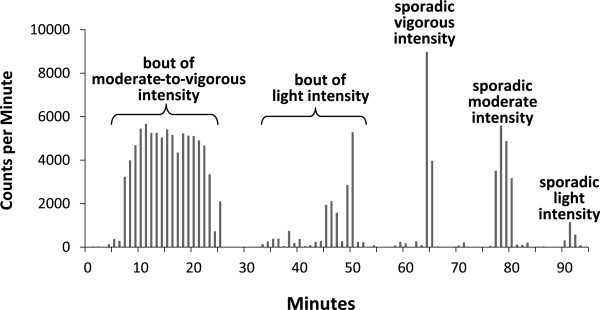
**Classification of physical activity data from 95 minutes of accelerometer data obtained on a 24-year-old man.**

After removing invalid days and participants with an insufficient number of valid days from the accelerometer database, each minute of physical activity data was categorized into one of four intensities based on established cut-points for the Actigraph AM-7164 accelerometer [20, 25]. Specifically, values between 0–99 counts per minute were classified as sedentary, values between 100–2019 were classified as light intensity, values between 2020–5998 were classified as moderate intensity, and values ≥ 5999 were classified as vigorous intensity. Data were then classified as being either BPA or SPA of different intensities, as explained in the following paragraph and as illustrated in Figure 1.

Initially, bouted MVPA was defined in the accelerometer database as periods of at least 10 consecutive minutes where the accelerometer counts exceeded the moderate threshold, with an allowance of 20% of the counts (e.g., 2 minutes for a 10 minute bout) being below the threshold [9, 10]. Once the 20% threshold was surpassed, the bout was stopped. The time spent in bouted MVPA was summed, a daily average was created, and this information was exported into a new dataset. Bouted MVPA was then removed from the accelerometer database. Next, light intensity BPA was defined, in what remained of the accelerometer database, as periods of at least 10 consecutive minutes where the accelerometer counts exceeded the light intensity threshold, with an allowance of 20% of the counts being below the threshold. Once the 20% threshold was surpassed, the bout was stopped. Note that light intensity BPA included MVPA, if the amount of MVPA did not satisfy the criteria for bouted MVPA. After light intensity BPA was counted, a daily average was created, and this information was exported into the new dataset. Light intensity BPA was then removed from the accelerometer database. At this phase, the accelerometer database only contained SPA and sedentary behavior. The daily averages for light intensity SPA, moderate intensity SPA, and vigorous intensity SPA were then calculated. These values were then exported in the new dataset. Total physical activity was then calculated in the new dataset as SPA + BPA. The new dataset was then merged with the other relevant NHANES datasets (eg, datasets that contained demographic information, BMI, etc.), and this merged dataset was use for the statistical analyses.

### Statistical analysis

Analyses were conducted using SAS v9.3 (SAS Institute Inc., Cary, NC) and SPSS v22 (IBM Corporation, Armonk, NY) and accounted for the clustered nature of NHANES and the adjusted sample weights that we created. Since the physical activity data were not normally distributed, we reported medians and interquartile ranges (IQR). Differences in median values across age, sex, ethnicity, and BMI groups were determined using Kruskal-Wallis omnibus and pairwise comparison tests. A p-value of < 0.05 was used to denote statistical significance. Bonferroni adjusted p-values were used when there were multiple pairwise comparisons (ie, p .05/number of pairwise comparisons).

## Results

A description of the sample is presented in Table 1. The majority of participants were between the ages of 20–30 and 40–59 years and of non-Hispanic white ethnicity. Approximately 32% were obese.

**Table 1:** **Characteristics of the study sample**

	Total		Men		Women	
Variable	Crude N	Weighted % (SE)	Crude N	Weighted % (SE)	Crude N	Weighted % (SE)
Age						
20-39 years	1672	37.3 (1.1)	904	39.8 (1.5)	768	35.0 (1.2)
40-59 years	1968	39.4 (1.0)	976	39.4 (1.3)	992	39.5 (1.2)
≥60 years	2400	23.3 (1.0)	1209	20.9 (1.1)	1191	25.5 (1.2)
Race/Ethnicity						
Non-Hispanic white	3241	72.2 (2.3)	1666	72.3 (2.3)	1575	72.1 (2.4)
Non-Hispanic black	1169	11.3 (1.4)	581	10.5 (1.3)	588	12.0 (1.5)
Mexican American	1226	7.7 (1.1)	650	8.6 (1.2)	576	6.8 (1.8)
Other	404	8.8 (0.8)	192	8.6 (0.9)	212	9.1 (0.9)
Body Mass Index						
<18.5 kg/m^2^	131	2.3 (0.2)	63	2.1 (0.3)	68	2.6 (0.3)
18.5-24.9 kg/m^2^	1767	31.8 (0.9)	826	26.7 (1.3)	941	36.6 (1.5)
25-29.9 kg/m^2^	2163	34.1 (0.9)	1283	40.3 (1.2)	880	28.1 (1.1)
≥30 kg/m^2^	1979	31.9 (1.1)	917	30.9 (1.5)	1062	32.8 (1.2)

Median daily minutes of SPA in the total sample is shown in Table 2. American adults spent 103 minutes/day (IQR: 85–121 minutes/day) engaging in SPA, which represented 27% (IQR: 19-36%) of their total (SPA + BPA) physical activity. The vast majority of SPA was of a light intensity (i.e., 100 of 103 minutes) and 91% of participants accumulated no vigorous intensity SPA. Since almost all group comparisons were statistically significant, the statistical significance of these differences is not highlighted in the remainder of the Results.

**Table 2:** **Median (interquartile range) daily time spent in sporadic physical activity of different intensities, in minutes per day and as a fraction of total of sporadic physical activity, within the total sample and according to sex, age, race/ethnicity, and body mass index**

	Minutes per day				Fraction of total physical activity			
	Light intensity	Moderate intensity	Vigorous intensity	All intensities	Light intensity	Moderate intensity	Vigorous intensity	All intensities
Total sample	100.4 (82.8-117.8)	2.1 (1.1-3.6)	0 (0–0)	103.0 (85.4-120.8)	26.1 (18.2-34.8)	0.5 (0.3-0.9)	0 (0–0)	26.9 (18.8-35.7)
Sex								
Men	97.6 (79.8-114.5)	2.6 (1.4-4.5)	0 (0–0)	100.9 (82.7-118.3)	24.7 (16.8-34.0)	0.6 (0.4-1.1)	0 (0–0)	25.6 (17.4-35.2)
Women	102.9 (86.0-121.0)	1.6 (0.9-2.8)	0 (0–0)	105.2 (88.6-124.1)	27.3 (19.6-35.5)	0.4 (0.2-0.7)	0 (0–0)	27.7 (20.0-36.2)
Age								
20-39 years	97.6 (78.3-114.8)	2.8 (1.7-4.6)	0 (0–0)	100.9 (81.7-118.4)	23.4 (15.7-31.6)	0.7 (0.4-1.3)	0 (0–0)	24.3 (16.3-32.9)
40-59 years	101.4 (84.5-119.8)	2.3 (1.3-3.6)	0 (0–0)	104.5 (87.2-123.5)	25.1 (18.1-33.0)	0.5 (0.3-0.9)	0 (0–0)	25.9 (18.6-34.0)
≥60 years	102.3 (87.3-119.1)	0.9 (0.5-1.6)	0 (0–0)	103.7 (88.4-120.5)	32.0 (23.8-44.4)	0.3 (0.2-0.5)	0 (0–0)	32.6 (24.2-44.9)
Race/Ethnicity								
Non-Hispanic white	100.6 (83.8-117.7)	2.0 (1.0-3.7)	0 (0–0)	103.4 (86.4-120.7)	26.7 (19.0-35.3)	0.5 (0.3-0.9)	0 (0–0)	27.4 (19.6-36.2)
Non-Hispanic black	102.8 (84.8-120.1)	2.0 (1.2-3.3)	0 (0–0)	105.3 (87.4-123.1)	25.8 (18.3-34.8)	0.5 (0.3-0.8)	0 (0–0)	26.4 (18.8-35.7)
Mexican American	92.0 (69.1-111.9)	2.2 (1.3-3.6)	0 (0–0)	94.6 (71.7-115.9)	19.9 (12.7-29.7)	0.5 (0.3-0.8)	0 (0–0)	20.6 (13.1-30.6)
Other	101.4 (80.8-122.9)	2.2 (1.3-3.5)	0 (0–0)	104.4 (84.8-125.7)	26.3 (16.7-35.6)	0.5 (0.3-0.9)	0 (0–0)	26.9 (17.1-36.5)
Body Mass Index								
<18.5 kg/m^2^	107.8 (84.6-121.7)	2.2 (1.2-4.4)	0 (0–0)	110.4 (90.7-126.4)	30.2 (21.9-41.3)	0.7 (0.4-1.1)	0 (0–0)	31.3 (22.6-43.1)
18.5-24.9 kg/m^2^	100.7 (82.6-119.0)	2.3 (1.2-3.9)	0 (0–0)	103.3 (85.6-122.0)	25.4 (17.4-33.4)	0.5 (0.3-0.9)	0 (0–0)	26.3 (18.1-34.5)
25-29.9 kg/m^2^	100.0 (83.3-117.2)	2.1 (1.0-3.7)	0 (0–0)	103.0 (86.2-120.8)	25.8 (18.2-34.3)	0.5 (0.3-0.9)	0 (0–0)	26.4 (18.7-35.5)
≥30 kg/m^2^	99.5 (82.3-116.8)	1.9 (1.1-3.2)	0 (0–0)	102.2 (84.4-119.6)	26.7 (18.8-36.4)	0.5 (0.3-0.8)	0 (0–0)	27.4 (19.2-37.3)

Note: All group comparisons were statistically significant.

Women accumulated slightly more SPA than men, both in minutes per day (105 vs. 101 minutes/day) and when expressed as a fraction of total physical activity (28 vs. 26%). The median minute per day SPA values were 3 to 4 minutes lower in 20–39 year olds than in the older two age groups. However in 60+ year olds, SPA as a fraction of total physical activity was 7 to 8 percentage points higher than in the two younger age groups. Mexican Americans spent 9 to 11 minutes/day less in SPA than non-Hispanic blacks and non-Hispanic whites. SPA did not differ in a meaningful way across normal weight, overweight, and obese groups; however, the underweight group accumulated 7 to 8 minutes/day more SPA than the other BMI groups. The age, race/ethnicity, and BMI patterns noted above were almost entirely attributable to differences in light intensity SPA. These patterns were also consistent when the sample was divided into men (Table 3) and women (Table 4).

**Table 3:** **Median (interquartile range) daily time spent in sporadic physical activity of different intensities, in minutes per day and as a fraction of total physical activity, within men and according to sex, age, race/ethnicity, and body mass index**

	Minutes per day				Fraction of total physical activity (SPA + BPA)			
	Light intensity	Moderate intensity	Vigorous intensity	All intensities	Light intensity	Moderate intensity	Vigorous intensity	All intensities
All Men	97.6 (79.8-114.5)	2.6 (1.4-4.5)	0 (0–0)	100.9 (82.7-118.3)	24.7 (16.8-34.0)	0.6 (0.4-1.1)	0 (0–0)	25.6 (17.4-35.2)
Age								
20-39 years	93.4 (72.6-110.7)	3.4 (2.0-5.3)	0 (0–0)	97.9 (76.8-115.8)	21.4 (13.8-30.0)	0.8 (0.4-1.3)	0 (0–0)	22.2 (14.3-31.1)
40-59 years	99.5 (80.8-116.6)	2.8 (1.8-4.6)	0 (0–0)	102.9 (83.8-121.2)	24.2 (16.8-32.6)	0.7 (0.4-1.2)	0 (0–0)	25.0 (17.5-33.8)
≥60 years	101.3 (86.1-117.1)	1.3 (0.6-2.0)	0 (0–0)	103.0 (87.5-118.9)	31.7 (24.2-44.5)	0.4 (0.2-0.7)	0 (0–0)	32.3 (24.7-45.1)
Race/Ethnicity								
Non-Hispanic white	98.4 (81.6-114.7)	2.5 (1.4-4.5)	0 (0–0)	101.5 (84.2-118.3)	25.7 (17.6-34.8)	0.6 (0.4-1.1)	0 (0–0)	26.7 (18.4-36.0)
Non-Hispanic black	100.8 (83.8-118.7)	2.5 (1.5-4.2)	0 (0–0)	103.9 (86.8-120.7)	23.5 (17.2-32.3)	0.6 (0.3-1.0)	0 (0–0)	24.4 (17.6-33.4)
Mexican American	82.0 (61.2-105.2)	2.7 (1.6-4.0)	0 (0–0)	86.2 (64.9-108.1)	17.1 (10.8-25.8)	0.5 (0.3-0.8)	0 (0–0)	17.7 (11.3-26.6)
Other	97.5 (76.5-115.9)	3.0 (1.9-4.7)	0 (0–0)	102.1 (78.7-121.0)	24.9 (14.9-36.5)	0.7 (0.4-1.3)	0 (0–0)	25.9 (16.1-37.5)
Body Mass Index								
<18.5 kg/m^2^	109.8 (81.4-122.7)	2.8 (1.5-4.6)	0 (0–0)	114.0 (82.9-127.1)	30.3 (20.8-40.9)	0.8 (0.5-1.1)	0 (0–0)	31.3 (21.6-42.7)
18.5-24.9 kg/m^2^	98.2 (78.8-114.6)	2.9 (1.6-4.8)	0 (0–0)	101.3 (83.0-117.7)	23.4 (15.6-33.1)	0.7 (0.4-1.1)	0 (0–0)	24.3 (16.5-34.4)
25-29.9 kg/m^2^	97.5 (80.8-114.5)	2.6 (1.4-4.5)	0 (0–0)	100.8 (83.4-119.0)	24.4 (16.8-32.9)	0.6 (0.4-1.1)	0 (0–0)	25.4 (17.4-34.2)
≥30 kg/m^2^	97.2 (79.1-113.8)	2.4 (1.4-4.0)	0 (0–0)	100.3 (81.7-117.3)	25.6 (17.5-35.6)	0.6 (0.3-1.1)	0 (0–0)	26.4 (18.3-36.7)

Note: All group comparisons were statistically significant.

**Table 4:** **Median (interquartile range) daily time spent in sporadic physical activity of different intensities, in minutes per day and as a fraction of total physical activity, within women and according to sex, age, race/ethnicity, and body mass index**

	Minutes per day				Fraction of total physical activity			
	Light intensity	Moderate intensity	Vigorous intensity	All intensities	Light intensity	Moderate intensity	Vigorous intensity	All intensities
All Women	102.9 (86.0-121.0)	1.6 (0.9-2.8)	0 (0–0)	105.2 (88.6-124.1)	27.3 (19.6-35.5)	0.4 (0.2-0.7)	0 (0–0)	27.7 (20.0-36.2)
Age								
20-39 years	102.0 (83.8-119.2)	2.4 (1.4-3.9)	0 (0–0)	104.6 (86.2-122.7)	25.7 (17.7-33.0)	0.6 (0.3-1.0)	0 (0–0)	26.6 (18.4-33.8)
40-59 years	103.4 (87.0-123.1)	1.8 (1.1-2.8)	0 (0–0)	105.6 (89.4-126.3)	25.7 (19.0-33.4)	0.4 (0.3-0.7)	0 (0–0)	26.5 (19.4-34.2)
≥60 years	103.6 (88.1-120.2)	0.8 (0.4-1.3)	0 (0–0)	104.7 (89.1-121.4)	32.4 (23.5-44.3)	0.2 (0.1-0.5)	0 (0–0)	32.7 (23.7-44.6)
Race/Ethnicity								
Non-Hispanic white	102.8 (86.4-120.6)	1.6 (0.8-2.9)	0 (0–0)	105.0 (89.3-123.1)	27.5 (20.1-35.6)	0.4 (0.2-0.7)	0 (0–0)	28.2 (20.6-36.2)
Non-Hispanic black	104.1 (86.0-121.0)	1.6 (0.9-2.6)	0 (0–0)	106.7 (88.2-124.7)	27.0 (19.8-35.9)	0.4 (0.2-0.7)	0 (0–0)	27.6 (20.1-36.7)
Mexican American	98.1 (79.8-118.5)	1.7 (1.0-2.7)	0 (0–0)	100.1 (82.0-120.9)	24.5 (16.2-32.5)	0.4 (0.2-0.7)	0 (0–0)	25.0 (16.6-33.7)
Other	104.6 (85.6-126.8)	1.7 (1.0-2.6)	0 (0–0)	105.9 (87.3-129.6)	26.7 (17.1-34.5)	0.4 (0.3-0.6)	0 (0–0)	27.1 (17.3-35.5)
Body Mass Index								
<18.5 kg/m^2^	106.1 (85.5-121.1)	2.0 (1.0-3.6)	0 (0–0)	108.3 (90.9-125.6)	29.8 (22.8-42.3)	0.6 (0.3-1.0)	0 (0–0)	31.1 (23.0-43.0)
18.5-24.9 kg/m^2^	102.1 (85.2-121.6)	1.8 (0.9-3.0)	0 (0–0)	104.7 (87.6-125.3)	26.7 (19.1-33.7)	0.4 (0.2-0.8)	0 (0–0)	27.3 (19.5-34.5)
25-29.9 kg/m^2^	103.6 (88.7-122.2)	1.4 (0.8-2.6)	0 (0–0)	106.0 (90.2-124.1)	27.1 (19.4-35.9)	0.4 (0.2-0.7)	0 (0–0)	27.7 (19.9-36.7)
≥30 kg/m^2^	102.9 (85.8-119.2)	1.6 (0.9-2.6)	0 (0–0)	104.6 (87.5-121.2)	27.9 (19.9-36.8)	0.4 (0.3-0.7)	0 (0–0)	28.6 (20.3-37.4)

Note: All group comparisons were statistically significant with the exception of vigorous intensity minutes in the Non-Hispanic black and Mexican American groups and light intensity minutes in the <18.5 kg/m^2^ and ≥30 kg/m^2^ groups.

Given the lack of moderate and vigorous intensity SPA, we decided *a posteriori* to investigate moderate and vigorous intensity activity embedded within light intensity BPA. This is hereafter referred to as embedded MVPA. Visual inspection of the accelerometer data revealed that many of the light intensity bouts contained some embedded MVPA (see Figure 1 for example). As shown in Table 5, the median value for embedded MVPA within the total sample was 16 minutes/day (IQR: 6–30 minutes/day). Men accumulated twice as much embedded MVPA as women (20 vs. 10 minutes/day) and 20–39 year olds accumulated more embedded MVPA than 40–59 year olds and 60+ year olds. Non-Hispanic blacks accumulated the least amount of embedded MVPA. Median embedded MVPA values were 5 to 8 minutes/day higher in the normal weight and overweight groups than in the underweight and obese groups.

**Table 5:** **Median (interquartile range) minutes per day spent in moderate-to- vigorous intensity physical activity embedded within bouts of light intensity physical activity within the total sample and according to sex, age, race/ethnicity, and body mass index**

	Moderate intensity	Vigorous intensity	Moderate-to-vigorous intensity
Total Sample	14.4 (5.2-28.5)	0.0 (0.0-0.2)	15.5 (5.7-29.7)
Sex			
Men	19.8 (8.3-36.2)	0.0 (0.0-0.4)	20.3 (8.5-38.2)
Women	9.7 (3.3-20.8)	0.0 (0.0-0.0)	9.8 (3.4-22.0)
Age			
20-39 years	20.5 (10.3-34.5)	0.0 (0.0-0.7)	21.2 (10.8-37.0)
40-59 years	15.4 (6.5-28.8)	0.0 (0.0-0.2)	15.9 (6.7-29.7)
≥60 years	3.3 (0.7-11.4)	0.0 (0.0-0.0)	3.3 (0.7-11.4)
Race/Ethnicity			
Non-Hispanic white	14.0 (4.7-28.0)^*a*^	0.0 (0.0-0.2)^*a*^	14.6 (4.8-29.4)^*a*^
Non-Hispanic black	12.6 (4.8-26.2)^*a*^	0.0 (0.0-0.2)^*a*^	12.7 (4.9-27.6)^*a*^
Mexican American	18.8 (8.4-37.0)^*b*^	0.0 (0.0-0.3)^*a*^	19.2 (8.5-38.0)^*b*^
Other	15.1 (6.6-29.0)^*b*^	0.0 (0.0-0.2)^*a*^	15.3 (6.7-30.7)^*b*^
Body Mass Index			
<18.5 kg/m^2^	12.0 (4.8-23.5)^*a*^	0.0 (0.0-0.2)^*ab*^	12.2 (4.8-24.0)^*a*^
18.5-24.9 kg/m^2^	17.1 (6.6-33.2)^*b*^	0.0 (0.0-0.6)^*b*^	18.3 (6.8-35.3)^*b*^
25-29.9 kg/m^2^	16.0 (5.8-29.7)^*b*^	0.0 (0.0-0.2)^*a*^	16.7 (5.9-30.9)^*b*^
≥30 kg kg/m^2^	10.2 (3.5-21.7)^*a*^	0.0 (0.0-0.0)^*a*^	10.3 (3.5-22)^*a*^

Note: All group comparisons were statistically significant unless the median (interquartile range) is followed by an ^a^or ^b^symbol. Groups with the same letter symbols were not statistically different from each other.

## Discussion

This representative sample of American adults spent 103 minutes/day engaging in SPA of any intensity, which represented 27% of their total (SPA + BPA) physical activity. Only 3 minutes/day of the SPA was of a moderate-to-vigorous intensity; however, the sample accumulated another 16 minutes/day of MVPA embedded within bouts of primarily light intensity activity. Although there were statistically significant differences in SPA across age, sex, ethnicity and BMI groups; these differences were small and primarily due to light intensity SPA.

Our finding that moderate-to-vigorous SPA represents 13% of total MVPA in American adults is lower than what has been reported previously. Within the Third Generation Cohort of the Framingham Heart Study, mean sporadic MVPA was 19 minutes/day, which represented 68% of total MVPA time [9]. Within a representative sample of Canadian adults, sporadic MVPA accounted for 53% of total MVPA energy expenditure [10]. In a representative sample of youth [14] and a small convenience sample of older adults [15] sporadic MVPA accounted for 66% of total MVPA time. The difference between our findings and previous findings is likely explained in large measure by differences in how SPA was classified. Specifically, in previous studies all MVPA occurring outside of bouted MVPA was considered sporadic. Conversely, in our study we calculated two forms of non-bouted physical MVPA; MVPA that was truly sporadic and MVPA that was embedded within bouts of primarily light intensity activity. While the truly sporadic MVPA only accounted for 2 minutes/day or 13% of total MVPA, the truly sporadic and embedded MVPA combined accounted for 17 minutes/day or 98% of total MVPA in the typical participant.

We feel that it is important to differentiate between MVPA that is truly sporadic and MVPA that is embedded within bouts of primarily light intensity activity as they may influence health differently and act through different biological mechanisms. True SPA may play a role in breaking up extended sedentary periods. Recent studies have shown that the frequency of breaks in sedentary periods is associated with cardiometabolic risk factors [25–27]. Conversely, MVPA embedded within light intensity bouts may impact health primarily by increasing energy expenditure. As the majority of American adults do not engage in sports [28, 29] that are known to contain a mixture of different movement intensities [30], it is likely that a major source of embedded MVPA in adults is through occupational activity. For example, a farmer may spend a large amount of time in light intensity activity walking around the farm then perform short bursts of higher intensity activity in the form of animal tending or hay bailing.

It is encouraging that the typical American adult accumulates 17 minutes/day of non-bouted MVPA, particularly given that they spend an average of <10 min/day in bouted MVPA [20], a volume of MVPA that falls well below that recommended for good health [3–5]. Non-bouted or SPA may also represent another intervention opportunity. SPA may be easier to intervene upon in populations without the free time to participate in BPA. That is, it may be easier for people to add a few minutes of physical activity here and there throughout the day rather than devoting a single longer block of time (eg, 30 minutes) to getting their activity. Also, SPA may provide an intervention opportunity for people who do not have the confidence or belief in their ability to participate in extended BPA. Doing a little bit of activity here and there may be perceived as being easier and less intimidating than bouts of activity. SPA based interventions also may have potential for greater maintenance of physical activity behavior post intervention, which is a known problem for BPA interventions [31].

Key limitations of this study warrant recognition. The physical activity patterns observed in this sample from 2003–2006 may not represent current behavior. While accelerometers provide an objective measure of physical activity, they are not without fault. They do not accurately capture activities that are not step based (i.e. cycling, swimming, upper body activities), and the accelerometers used in NHANES were uniaxial and primarily captured horizontal movement at the hip. Nonetheless, we feel that a vast majority of physical activity was captured since walking is the most common form of leisure-time physical activity [32] and is heavily involved in occupational and transportation activities. Conversely, less than 2% of Americans engage in strength training and cycling [28].

## Conclusion

SPA of any intensity accounted for 27% of total daily physical activity in Americans adults. Sporadic MVPA and MVPA embedded within light intensity bouts accounted for 98% of total MVPA. Future research is needed to better understand the types of activity that represent embedded MVPA patterns, and to investigate if sporadic MVPA and embedded MVPA have a different impact on health outcomes.
